# Acute myocardial infarction caused by vasospasm of a jailed diagonal branch subsequent to stent implantation in the left anterior descending artery: a case report

**DOI:** 10.1093/ehjcr/ytae421

**Published:** 2024-08-22

**Authors:** Hiroshi Yoshikawa, Tomoyo Sugiyama, Makoto Araki, Taishi Yonetsu, Tetsuo Sasano

**Affiliations:** Department of Cardiovascular Medicine, Tokyo Medical and Dental University, 1-5-45, Yushima, Bunkyo Ward, Tokyo 113-8519, Japan; Department of Cardiovascular Medicine, Tokyo Medical and Dental University, 1-5-45, Yushima, Bunkyo Ward, Tokyo 113-8519, Japan; Department of Cardiovascular Medicine, Tokyo Medical and Dental University, 1-5-45, Yushima, Bunkyo Ward, Tokyo 113-8519, Japan; Department of Cardiovascular Medicine, Tokyo Medical and Dental University, 1-5-45, Yushima, Bunkyo Ward, Tokyo 113-8519, Japan; Department of Cardiovascular Medicine, Tokyo Medical and Dental University, 1-5-45, Yushima, Bunkyo Ward, Tokyo 113-8519, Japan

**Keywords:** Case report, Angina, Coronary spasm, Stent-jailed side branch

## Abstract

**Background:**

Coronary stents have been reported to cause endothelial dysfunction, potentially leading to spasm at the edges of the stent. However, the clinical significance of vascular spasm in stent-jailed side branches remains poorly understood.

**Case summary:**

A 67-year-old woman was referred to our hospital for angina occurring both during exercise and at rest. An everolimus-eluting stent was implanted for a physiologically significant stenosis in the proximal left anterior descending artery, while an intermediate stenosis persisted in the jailed first diagonal branch. Although her exertional angina resolved, her rest symptoms worsened after percutaneous coronary intervention (PCI). She was admitted with acute myocardial infarction 1 month later. Urgent coronary angiography showed no stent failure, but an acetylcholine provocation test induced a spasm leading to total occlusion of the jailed diagonal branch. An additional stent was implanted in the diagonal branch due to a residual stenosis even after isosorbide dinitrate administration. After the second PCI, her chest pain completely resolved.

**Discussion:**

This is the first documentation of aggregated coronary spasm observed at the ostium of stent-jailed side branch. Stent implantation may induce endothelial dysfunction and promote inflammation, leading to spasms particularly at stent edges. This phenomenon can extend to side branches jailed by the stent, and invasive intervention may be a viable therapeutic strategy for such cases.

**Learning points**

- Coronary spasms following stent implantation may occur not only in stent edges but also in jailed side branches.
- For the management of stent-induced spasm in a side branch, percutaneous coronary intervention may be considered, particularly in the presence of organic stenosis in the side branch.

## Introduction

Approximately 20–40% of patients may have persistence or recurrence of angina despite successful percutaneous coronary interventions (PCIs).^1^ One of the possible causes of post-PCI angina is impaired vasomotion of coronary arteries following stent implantation.^1^ Previous studies have shown that coronary stents may induce endothelial dysfunction and spasms along the stented sites.^2–5^ However, these investigations primarily concentrated on spasms at the edges of stents, with no documented evidence concerning spasms in jailed side branches. Herein, we present a case of acute myocardial infarction caused by a spasm in a jailed diagonal branch following stent implantation in the left anterior descending artery (LAD).

## Summary figure

**Table:** 

Initial presentation	A 67-year-old woman was referred to our hospital for chest pain.
1 month later	Coronary computed tomography angiography revealed an intermediate stenosis in the proximal LAD.
2 months later	Due to positive fractional flow reserve (FFR), an everolimus-eluting stent was implanted to the proximal LAD, while 50% ostial stenosis remained at the jailed diagonal branch.
3 months later	She was urgently hospitalized with a diagnosis of myocardial infarction. An acetylcholine provocation test induced a spasm of the jailed diagonal branch. Percutaneous coronary intervention for the diagonal branch was performed with implantation of an everolimus-eluting stent.
4 months later	She has been free from symptoms during follow-up.

## Case presentation

**Figure 1:**
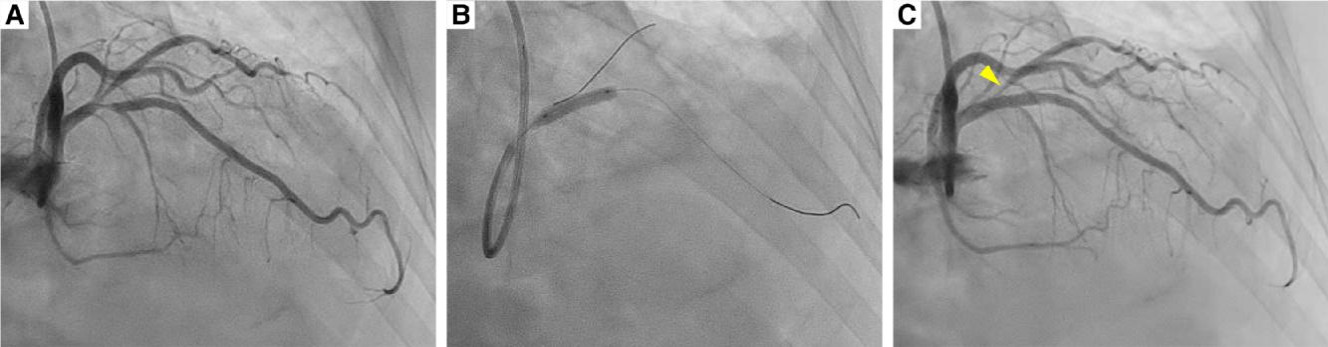
Initial percutaneous coronary intervention. (*A*) Pre-intervention left coronary angiogram. (*B*) An everolimus-eluting stent was implanted in the proximal left anterior descending artery. (*C*) Post-intervention left coronary angiogram. Fifty per cent ostial stenosis remained in the jailed first diagonal branch (Arrowhead).

**Figure 2:**
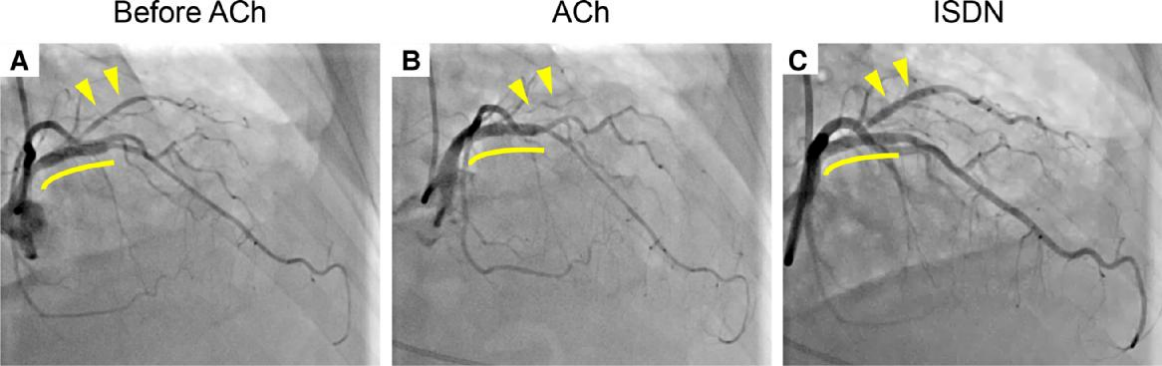
Acetylcholine provocation test prior to the second percutaneous coronary intervention. (*A*) Left coronary angiogram before acetylcholine administration. (*B*) Intracoronary acetylcholine-induced complete occlusion at the ostium of the jailed first diagonal branch. (*C*) Intracoronary isosorbide dinitrate reopened the jailed branch. Curved lines indicate the everolimus-eluting stent in the proximal left anterior descending artery. Arrowheads indicate the jailed first diagonal branch.

**Figure 3:**
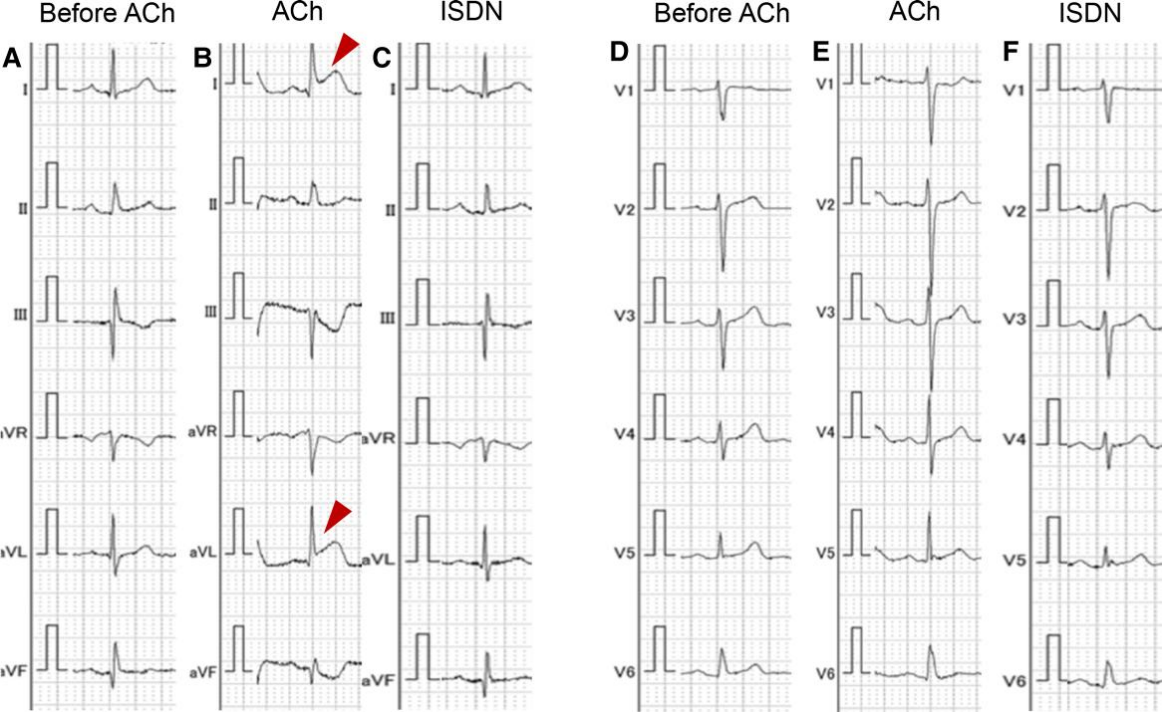
Electrocardiographic changes during acetylcholine provocation test. Acetylcholine provocation test induced ST-segment elevation in leads I and aVL (Arrowheads). (A and *D*) Electrocardiogram before acetylcholine administration. (*B* and *E*) Electrocardiogram after intracoronary acetylcholine administration. (*C* and *F*) Electrocardiogram after intracoronary isosorbide dinitrate administration.

**Figure 4:**
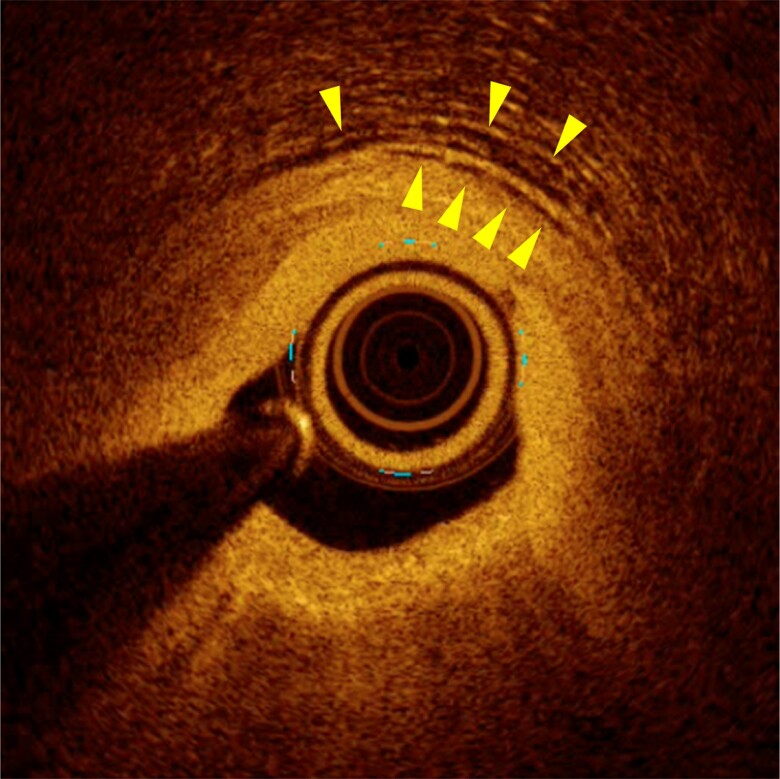
Pre-intervention optical coherence tomography image of the jailed first diagonal branch on the second percutaneous coronary intervention. A lipidic plaque and enhanced vasa vasorum formation (Arrowheads) were shown in the adventitia around the ostium of the first diagonal branch.

**Figure 5:**
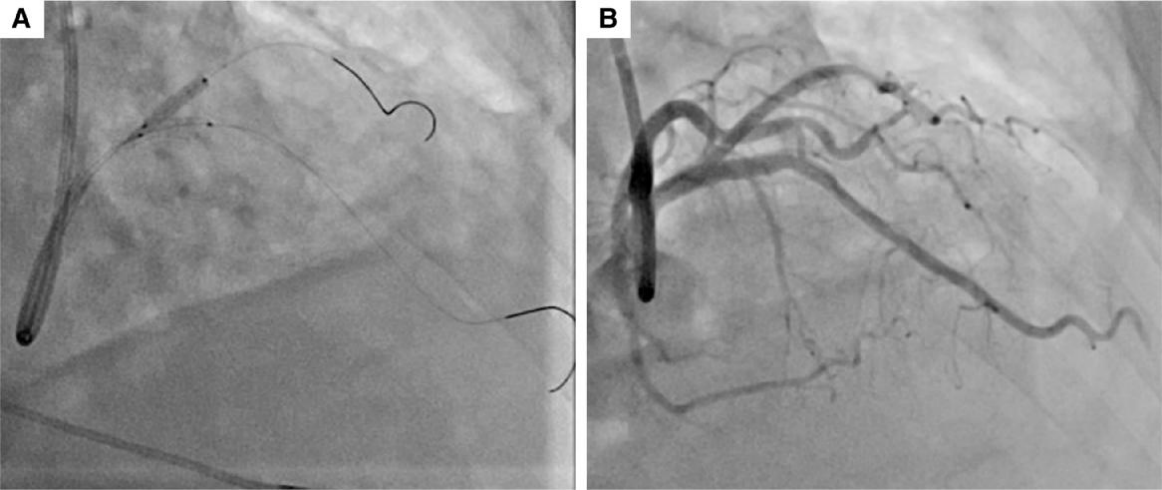
Second percutaneous coronary intervention. (*A*) An everolimus-eluting stent was implanted in the first diagonal branch, followed by balloon dilatation inside the stent in the main branch of the left anterior descending artery. (*B*) Left coronary angiograms after the second percutaneous coronary intervention.

A 67-year-old woman was referred to our hospital for chest pain lasting for 2 months that occurred both during exercise and at rest. She had a history of interstitial pneumonia, which had required home oxygen therapy. Coronary computed tomography angiography showed an intermediate stenosis in the proximal segment of the LAD. Subsequent coronary angiography confirmed a significant stenosis in the proximal LAD with a positive FFR of 0.78 (*Figure 1A*). Percutaneous coronary intervention was successfully performed, implanting a 3.5 × 15 mm everolimus-eluting stent in the main branch of the LAD (*Figure 1B*). Although the ostium of the first diagonal branch jailed by the stent showed a residual 50% stenosis, additional procedure was deferred without performing physiological assessment since the lumen diameter and coronary blood flow were apparently preserved (*Figure 1C*). After the initial PCI, her chest pain on exertion was resolved, while her rest symptoms became more frequent particularly at night and in early morning, even after initiation of a calcium channel blocker. At the scheduled outpatient visit at 1 month after PCI, her high-sensitivity cardiac troponin I was elevated (781 pg/mL) despite of unchanged electrocardiograms. She was diagnosed with non–ST-segment elevation myocardial infarction and underwent urgent coronary angiography. Coronary angiogram showed no in-stent restenosis in the LAD or progression of ostial stenosis of the jailed first diagonal branch (*Figure 2A*). Given her symptoms accumulated at rest, vasospastic angina was strongly suspected, and an acetylcholine provocation test was precedingly performed to clarify the involvement of vasospasm. Shortly after administration of 50 µg acetylcholine to the left coronary artery, her chest pain was reproduced accompanying ST-segment elevation in leads I and aVL (*Figure 3*). Coronary angiogram showed total occlusion at the ostium of the first diagonal branch. Although her coronary arteries appeared diffusely narrowed, high-grade stenosis > 70% was not induced in other segments of the epicardial coronary arteries (*Figure 2B*). After the administration of isosorbide dinitrate (ISDN) to the left coronary artery, occlusion of the first diagonal branch, chest pain, and ST-segment elevation were all resolved (*Figure 2C*). Although vasospasm was almost completely resolved by the administration of ISDN, there was a remaining intermediate organic stenosis at the ostium of the first diagonal branch which indicated positive for ischaemia with an FFR of 0.65. Careful discussion was made on whether to revascularize the pinched diagonal branch or leave it alone. However, because of her frequent chest pain despite the daily use of a calcium channel blocker, a decision was made to perform PCI. Optical coherence tomography showed a lipidic plaque and enhanced vasa vasorum formation around the adventitia at the ostium of the first diagonal branch (*Figure 4*). A 2.25 × 12 mm everolimus-eluting stent was implanted in the first diagonal branch, followed by balloon dilatation inside the stent in the main branch of the LAD to crush the protruding proximal edge of the stent in the diagonal branch (*Figure 5*). After discharge, her chest pain completely resolved.

## Discussion

Coronary spasms of the stented vessels can cause persistent or recurrent angina post-PCI.^1^ Several studies reported the incidence of stent-edge spasm induced by acetylcholine provocation tests after stent implantation, which varied according to the type of stents: 14% with bare metal stents,^2^ 85.7% with the first-generation drug-eluting stents (DESs),^3^ and 19.5% with the second-generation DES.^6^ Those studies indicated that stent-related spasm is not rare and remains a significant concern.

The pathogenesis of stent-induced coronary hyperconstriction is the enhancement of vasa vasorum formation and inflammation in the adventitia extending from stented sites to stent edges, which lead to Rho-kinase activation.^7,8^ Another study suggested that the polymer component of DES may play an important role in the mechanisms of coronary inflammation and endothelial dysfunction.^9^ It is plausible that those mechanisms could be present not only at stent edges but also in jailed side branches, as shown in the present case. In addition, coronary microvascular dysfunction (CMD) could have contributed to her angina. In fact, coronary flow reserve was 1.3 and the index of microcirculatory resistance was 40 at the diagonal branch, which represented significantly impaired microvascular function in that territory. It has been reported that patients with vasospastic angina have endothelium-dependent CMD,^10^ and approximately 23% of patients with ischaemia with non-obstructive coronary arteries have both vasospastic angina and CMD.^11^ Suda *et al*.^12^ reported that Rho-kinase activation was a common mechanism for the development of both vasospasm and CMD. Therefore, it is plausible that the stent implanted in the LAD may have induced CMD as well as vasospasm in the diagonal branch.

In this case, the combined involvement of vasospasm and organic stenosis at the first presentation cannot be ruled out, since the patient initially presented with concurrent chest pain on exertion and at rest. However, considering that her resting angina was significantly exacerbated after the index PCI for LAD, even with the initiation of a calcium channel blocker, and the fact that a significant spasm leading to total vessel occlusion was induced only in the diagonal branch while no significant stenosis was provoked in the other coronary vessels, we surmised that the stent implantation in the LAD may have induced spasm in the jailed diagonal branch.

For the management of stent-induced coronary vasomotion abnormalities, calcium channel blockers, such as long-acting nifedipine, are effective due to their vasodilation and anti-inflammatory effect.^6^ Nevertheless, in the present case, chest pain at rest was rather aggravated after the initial PCI despite the initiation of a calcium channel blocker, and the symptoms were completely resolved after the second PCI to the jailed diagonal branch. This could be attributed to the fact that if the jailed side branch originally has a significant organic stenosis as in our case, the vessel lumen is readily occluded even with low- or moderate-grade spasms occurring in the side branch. Furthermore, it should be noted that particularly after stent implantation of a main vessel, carina shift is likely to induce organic stenosis in the jailed side branch.^13^ In summary, ischaemia in stent-jailed branches could be attributed to both stent-induced spasm and organic stenosis which originally presents or is induced due to carina shift. Our case suggested that pharmacotherapy alone may not adequately work to manage the symptoms; and therefore, invasive interventions may be a therapeutic option for the treatment of spasm in stent-jailed branch.

## Data Availability

The data underlying this article are available in the article and in its online Supplementary material.

## Lead author biography

Hiroshi Yoshikawa graduated from Yokohama City University and completed the MD course in 2018. He is a graduate student at the Department of Cardiovascular Medicine at Tokyo Medical and Dental University. He is currently working as a cardiovascular interventional fellow at Hiratsuka Kyosai Hospital.

## Notes

**Consent:** The patient has given written informed consent for publication in accordance with the COPE guidelines.

**Funding:** None declared.
